# A Feasibility Study of Tablet-Based Eye Movement Assessment Using a Built-In Camera: A Pilot Study

**DOI:** 10.3390/jemr19020024

**Published:** 2026-02-24

**Authors:** Kyunghyun Park, Unseok Lee, Sejoon Moon, Hyungsik Bae, Hyungoo Kang

**Affiliations:** 1 Department of Optometry, Seoul National University of Science and Technology, Seoul 01811, Republic of Korea; 2 Department Biomedical Laboratory Science, Catholic Kwandong University, Gangneung 25522, Republic of Korea; 3 Department of Optometry, Catholic Kwandong University, Gangneung 25522, Republic of Korea

**Keywords:** tablet PC, eye movement test, visual function, smooth pursuit eye movement, saccadic eye movement

## Abstract

This study developed a tablet PC–based eye movement assessment application and conducted a pilot investigation to explore whether tablet-based ocular motor metrics demonstrate functional sensitivity to variations in conventional visual function parameters. Twenty-three healthy adults (10 males, 13 females; mean age: 24.41 ± 1.91 years) without a history of ocular disease performed smooth pursuit and saccadic eye movement tests at three difficulty levels. For exploratory analysis, participants were stratified into above- and below-mean groups based on conventional visual function test results. For smooth pursuit movements, mean pursuit traversal time demonstrated statistically significant differences between the low–medium (1.11 s) and low–high (1.14 s) difficulty levels (*p* < 0.05), with corresponding differences in derived velocity. Saccadic movements showed significant mean accuracy differences between low-high (1.02 points) and medium-high (0.95 points) difficulty levels (*p* < 0.05). Participants with higher-than-average horizontal phoria values (distance and near) and the blur/break points of near convergence amplitude exhibited significantly longer smooth pursuit traversal times (corresponding to slower derived velocities) (*p* < 0.05). The high-value group for blur point of near convergence amplitude demonstrated significantly superior saccadic accuracy (1.63 points) compared with the low-value group (1.30 points) (*p* < 0.05). Exploratory associations between visual function parameters and ocular motor performance were observed within the healthy participant group, suggesting exploratory associations between tablet-based smooth pursuit and saccadic eye movement performance and conventional visual function measures. These findings suggest that tablet PC–based eye movement assessment may serve as a feasible, low-cost approach for exploratory screening and functional monitoring, rather than a validated diagnostic tool.

## 1. Introduction

The visual system conveys images from both eyes to the central nervous system, and precisely coordinated eye movements ensure stable and clear visual perception [1]. These movements fall into two main categories: Smooth Pursuit Eye Movements (SPEM), which smoothly follow moving targets to maintain their image on the fovea, and Saccadic Eye Movements (SEM), which are rapid shifts in gaze between fixation points [2]. As each type is controlled by distinct neural circuits, their speed, accuracy, and latency serve as key indicators of ocular and neurological health.

Eye-tracking technology, long established in cognitive neuroscience, enables quantitative assessment of these metrics to identify subtle visual or neurological dysfunctions often undetected by traditional methods [3]. It has proven valuable in evaluating disease severity and cognitive performance in Multiple Sclerosis (MS) [4], detecting visual deficits after mild Traumatic Brain Injury (mTBI) [5], and characterizing reading abnormalities in Dyslexia [6]. Recent integration of machine learning and deep learning algorithms with eye-tracking data has further improved diagnostic accuracy and pattern recognition [7].

Technological advances in mobile devices have made it possible to implement software-based eye tracking using front-facing cameras on smartphones and tablets [8]. Although these portable systems provide lower resolution than laboratory-grade equipment, they greatly enhance accessibility and scalability in screening applications [9]. This portability supports broader use in monitoring chronic disorders such as ADHD [10] and in acute care settings such as concussion assessment in sports medicine [11]. Research increasingly supports the use of tablet PCs for visual assessment and training [12]. For example, tablet-based smooth pursuit training has been shown to reduce hemispatial neglect symptoms in post-stroke patients [13], while computer-based eye movement programs have improved gaze control in children with ADHD [14].

While several tablet- and computer-based eye movement assessment programs have been previously reported, most existing approaches either rely on external eye-tracking hardware or focus on isolated eye movement metrics without systematic manipulation of task difficulty. In addition, many mobile eye-tracking studies primarily emphasize feasibility rather than functional differentiation of oculomotor performance [15,16,17].

The proposed system differs from prior work in three key aspects. First, it implements a fully software-based eye-tracking approach using only a single front-facing tablet camera, without requiring external sensors or wearable devices. Second, both smooth pursuit and saccadic eye movements are assessed within a unified application using predefined, graded difficulty levels designed to systematically challenge oculomotor control. Third, the system is designed not as a diagnostic replacement for laboratory-grade eye trackers, but as a low-cost, portable screening tool that can be used in non-laboratory environments.

Therefore, the purpose of this pilot study was to investigate the feasibility of a fully software-based, tablet PC–based eye movement assessment system and to explore whether graded task difficulty could capture relative differences in ocular motor performance in a healthy young adult population. This study does not aim to validate the system against laboratory-grade eye-tracking devices or to establish diagnostic criteria. Instead, it focuses on exploring whether tablet-based eye movement metrics show meaningful associations with conventional visual function measures, thereby providing preliminary evidence for functional sensitivity in non-laboratory settings.

## 2. Materials and Methods

### 2.1. Study Participants

The study included 23 healthy adult participants (10 males, 13 females) in their 20s, with a mean age of 24.17 ± 1.57 years. None of the participants had any known ocular disease. Eight participants wore glasses, nine wore contact lenses, and six had uncorrected vision. To minimize developmental and aging-related variability in ocular motor performance, the participant group was restricted to healthy young adults for this pilot feasibility study.

### 2.2. Ocular Motor Assessment System

**Figure 1:**
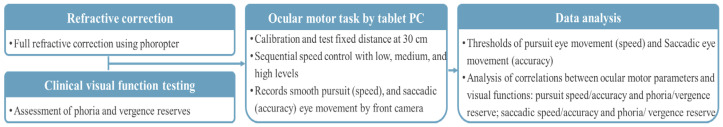
Flowchart of the experimental protocol, encompassing clinical pre-assessment, tablet-based ocular motor tasks, and subsequent data analysis.

The experimental workflow consists of a systematic three-stage process: clinical pre-assessment, tablet-based ocular motor tasks, and comprehensive data analysis (Figure 1). Initially, participants undergo full refractive correction using a phoropter, followed by the assessment of phoria and vergence reserves to establish baseline visual function.

Subsequently, saccadic and smooth pursuit eye movement tasks are performed on a tablet PC. For gaze measurement, we employed the Eyedid SDK API 2.6.1 (Visualcamp, Seoul, Republic of Korea), a software-based eye-tracking solution that utilizes the tablet’s integrated front-facing camera without supplementary hardware [18]. This SDK provides a robust tracking environment through rapid 5-point user-specific calibration and multi-platform compatibility, ensuring stable data acquisition. The SDK estimates gaze position based on facial and ocular features extracted from the tablet’s front-facing camera stream and outputs real-time two-dimensional gaze coordinates. However, the SDK does not expose numerical metrics for angular accuracy, spatial precision, or drift, which limits direct comparison with laboratory-grade eye-tracking systems. The system records the speed and accuracy of eye movements across three sequential difficulty levels (low, medium, and high). By leveraging the SDK’s real-time gaze estimation algorithms, the application captures high-resolution ocular motor data, which is then processed to extract quantitative metrics.

#### 2.2.1. Hardware and Stimulus Configuration

A tablet PC Galaxy Tab S8 Ultra (Samsung Electronics, Suwon, Republic of Korea) and a custom-developed application, Etrackxer (version 1.0; Optoreal, Republic of Korea), were used for ocular motor measurement. Eye movement data were obtained using the tablet’s built-in front-facing camera, enabling a fully software-based eye-tracking approach without external eye-tracking hardware. Gaze estimation was implemented using the Eyedid SDK API (version 2.6.1; Visualcamp, Republic of Korea), a commercial camera-based eye-tracking solution designed for mobile and tablet environments.

**Figure 2:**
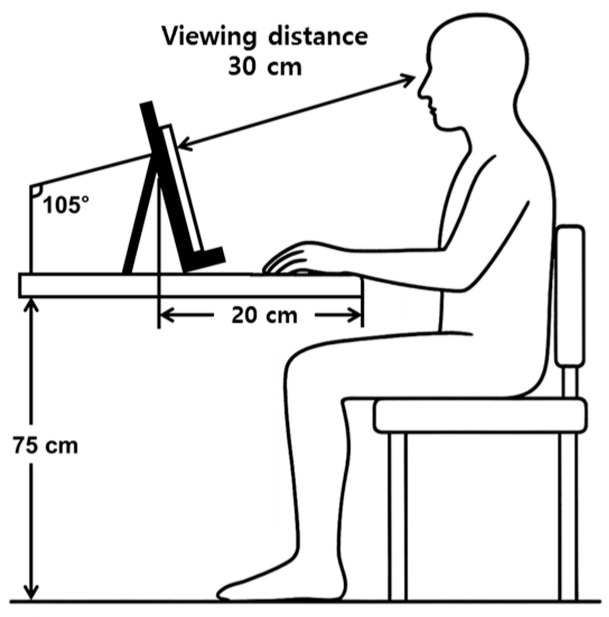
Schematic diagram of the experiment.

The tablet PC featured a 14.6-inch display (326.4 mm × 208.6 mm × 5.5 mm), and the viewing distance was standardized at 30 cm using a fixed stand (Figure 2). The moving target was displayed within a screen area of 25 cm (horizontal) × 16 cm (vertical), corresponding to a visual angle of approximately 45° horizontally and 30° vertically. The eye-tracking software operated at an effective gaze sampling rate of approximately 60 Hz, corresponding to the nominal camera frame rate under stable tracking conditions. Because sampling was implemented in a software-based environment, occasional dropped frames occurred during gaze loss or head movement; such samples were excluded during data screening.

Prior to task execution, a user-specific calibration procedure was performed using the Eyedid SDK’s built-in calibration framework. Calibration was conducted in five-point mode, in which participants were instructed to sequentially fixate on five predefined target locations distributed across the screen to ensure robust spatial coverage. The standard calibration accuracy criterion provided by the SDK was applied. During calibration, real-time calibration events (e.g., calibration progress, next-point prompts, and completion status) were monitored via the SDK’s event interface, allowing visual verification of calibration quality by the experimenter.

If gaze estimation appeared unstable or if frequent tracking state errors (e.g., loss of gaze detection or face tracking) were observed during calibration, the procedure was repeated before proceeding to the experimental tasks. The Eyedid SDK does not provide continuous numerical drift correction during task execution; therefore, calibration quality was maintained through initial verification and post hoc data screening. Measurements were excluded when participants moved beyond the measurable range, when gaze deviated outside the screen area, or when sustained tracking instability occurred.

Because the eye-tracking system used in this study is based on a commercial, software-only SDK designed for mobile environments, standard laboratory eye-tracking performance metrics—such as angular accuracy, spatial precision, and drift—were not directly accessible. Therefore, system performance was evaluated indirectly through calibration success, tracking stability during task execution, and post hoc data screening. The reported eye movement measures should be interpreted as functional gaze estimates rather than validated eye-tracking metrics. As such, the present study focuses on feasibility and functional sensitivity rather than measurement validation.

To minimize interface-related variability, all tasks were presented using a uniform, neutral background color with high contrast between the target and background. This system design was intended to enhance measurement reliability and robustness under real-world conditions beyond controlled laboratory environments.

#### 2.2.2. Eye Movement Testing Protocol

**Figure 3:**
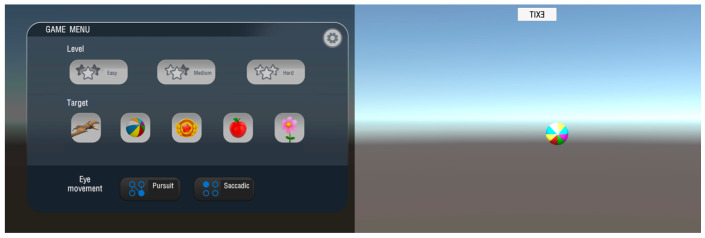
Application interface for the eye-movement test with integrated eye-tracking function.

The Etrackxer application comprised two types of ocular motor tasks—Smooth Pursuit Eye Movement (SPEM) and Saccadic Eye Movement (SEM)—each presented at three difficulty levels: low (Level 0), medium (Level 1), and high (Level 2) (Figure 3). Participants were instructed to fixate on the moving target displayed on the Tablet PC. The experimenter monitored participants for posture changes and fatigue throughout the session. The procedure began with SPEM trials (from low to high difficulty), followed by SEM trials in the same sequence. For smooth pursuit eye movements, task difficulty was manipulated by systematically increasing target velocity across the three levels, whereas for saccadic eye movements, difficulty was increased by reducing stimulus presentation time and tightening spatial accuracy thresholds. Prior to the formal testing session, participants completed a short familiarization trial to ensure understanding of the task instructions and to reduce learning effects during data collection. Rest periods were provided between task conditions to minimize fatigue-related performance degradation, particularly at higher difficulty levels. All tasks were presented using a uniform, neutral background color with high contrast between the target and background to minimize UI-induced variability.

#### 2.2.3. Smooth Pursuit Eye Movement (SPEM) Measurement

Smooth pursuit performance was quantified using average traversal time, defined as the time required for the gaze to complete ten full traversals of the target trajectory. The target moved horizontally back and forth 10 times, defined as the angular distance of the target trajectory divided by the time required for the gaze to complete ten full traversals of the trajectory. Specifically, the trajectory spanned a fixed angular distance of D degrees, and the average pursuit velocity (V) was calculated as: V=D/T where T denotes the average traversal time (in seconds). Target speeds were set at 53.07°/s for low (Level 0), 61.23°/s for medium (Level 1), and 72.36°/s for high (Level 2) difficulty. Instances in which gaze tracking was lost because the eyes left the measurement area were excluded from the average velocity calculations. Difficulty levels for smooth pursuit eye movements were defined by systematically increasing target velocity. Target speeds were selected based on previously reported physiological limits of smooth pursuit, which typically range between approximately 40°/s and 80°/s in healthy adults. The selected velocities were intended to progressively challenge pursuit stability while remaining within a safe and feasible range for a pilot study [19,20]. Gaze velocity was computed directly from successive gaze position samples provided by the eye-tracking SDK. No explicit filtering or segmentation was applied to separate smooth pursuit epochs from catch-up saccades; therefore, the reported velocity reflects combined gaze motion, including corrective saccades. This approach was adopted for feasibility testing in this pilot study and is acknowledged as a limitation. Accordingly, the reported pursuit velocity values should be interpreted as reflecting combined gaze motion rather than pure smooth pursuit velocity.

#### 2.2.4. Saccadic Eye Movement (SEM) Measurement

SEM accuracy was evaluated using an ordinal scoring system (0–2 points) based on a deviation metric defined as the normalized distance between the gaze endpoint and the target location at saccade completion. Lower deviation values indicated greater accuracy. Thresholds of ≤0.6 and ≤1.2 were used to assign scores of 2 and 1, respectively, while deviations > 1.2 were assigned a score of 0. Although mean SEM accuracy scores were analyzed in this pilot study for exploratory purposes, this metric should be interpreted as ordinal rather than continuous.

For saccadic eye movements, task difficulty was manipulated by reducing stimulus presentation time and tightening spatial accuracy thresholds. These parameters were designed to increase temporal and spatial demands on rapid gaze shifts, consistent with prior saccadic task designs reported in the literature [21,22].

#### 2.2.5. System Performance Considerations

The eye-tracking system employed in this study is based on a commercial, software-based SDK designed for mobile devices. Although the SDK provides real-time gaze coordinates and tracking state indicators, it does not expose explicit numerical metrics for spatial accuracy (e.g., mean angular error in degrees), precision, or continuous drift correction.

System performance was therefore evaluated indirectly through calibration success, tracking stability during tasks, and post hoc data screening. Trials were excluded if gaze tracking was lost for extended periods, if the face was not detected, or if gaze samples fell outside the display region. Under stable conditions, gaze data were sampled at an effective rate of approximately 60 Hz.

While these procedures ensured a consistent baseline level of data quality suitable for feasibility testing, the lack of quantified accuracy and precision metrics represents a limitation of the present study and underscores the need for future validation against laboratory-grade eye-tracking systems. Accordingly, the present system should be interpreted as providing functional gaze estimates rather than validated eye-tracking measurements, and the reported metrics should not be directly compared with laboratory-grade eye-tracking accuracy benchmarks.

### 2.3. Clinical Visual Function Testing

Clinical visual function tests were performed prior to ocular motor assessment using a phoropter HDR-9000 (Huvitz, Anyang, Republic of Korea), a device known for its accuracy, speed, and convenience in precision eye examinations.

#### 2.3.1. Refractive Correction and Full Correction

Objective refraction was initially assessed using auto refractometer (Huvitz, HRK-1, Republic of Korea). For participants wearing corrective lenses, measurements were taken after achieving full refractive correction with the phoropter. Refractive errors were refined using a radial chart and the Jackson Cross Cylinder (JCC) method, which adjusts both the axis and power of astigmatism. Following astigmatism correction and prism dissociation binocular balancing, full refractive power was verified using a trial frame.

#### 2.3.2. Measured Clinical Parameters

Distance vision was assessed at 6 m and near vision at 40 cm. Horizontal phoria was measured using the Modified von Graefe method. Rotary prisms were employed to measure the blur point, break point, and recovery point for convergence and divergence reserves by base-out and base-in prisms.

### 2.4. Statistical Analysis

All statistical analyses were conducted using SPSS version 28.0 (IBM Corp., Armonk, NY, USA). Given the relatively small sample size (*n* = 23), data normality was first assessed using the Shapiro–Wilk test. Depending on the normality results, either parametric or nonparametric methods were applied as appropriate. To evaluate differences in smooth pursuit eye movement (SPEM) mean traversal time (and corresponding derived velocity) and saccadic eye movement (SEM) accuracy across difficulty levels (low, medium, and high), a repeated-measures analysis of variance (ANOVA) was performed. Mauchly’s test of sphericity was used to assess the sphericity assumption, and when this assumption was violated, the Greenhouse–Geisser correction was applied to adjust the degrees of freedom. Post hoc pairwise comparisons with Bonferroni adjustment were conducted to identify significant differences between difficulty levels.

For analyses comparing performance, participants were grouped separately based on whether their overall mean SPEM velocity values were above or below the overall mean, and whether their overall mean SEM accuracy values were above or below the overall mean. Participant stratification into above- and below-mean groups was performed solely for exploratory visualization and trend identification. This approach was not intended to imply clinical categorization or diagnostic thresholds, but rather to facilitate preliminary interpretation in this pilot study. Independent-samples t-tests were used to compare visual functions such as phoria, vergence reserves, and accommodative functions between these groups. When data did not meet normality assumptions, Mann–Whitney U tests were employed instead. Statistical significance was set at *p* < 0.05. All statistical comparisons involving group stratification were conducted solely to explore potential trends and should not be interpreted as confirmatory or hypothesis-driven analyses. Because saccadic eye movement (SEM) accuracy was quantified using an ordinal scoring system (0–2 points), parametric analyses were applied for descriptive and exploratory purposes only. To verify the robustness of the observed trends, nonparametric analyses were additionally conducted where appropriate, yielding consistent directional patterns. Effect sizes and confidence intervals were not systematically reported due to the exploratory focus and limited sample size of this pilot study; future studies with larger samples will incorporate comprehensive effect size estimation. Nonparametric repeated-measures analyses (Friedman test) yielded consistent directional trends with the parametric results.

## 3. Results

For exploratory analysis only, participants were stratified into above- and below-mean groups based on visual function measures. This stratification was used solely to visualize potential trends and does not imply clinical categorization or diagnostic thresholds. All group-based analyses were conducted for exploratory purposes to visualize potential trends and were not intended to support inferential or population-level conclusions.

### 3.1. Smooth Pursuit Eye Movement Performance

**Figure 4:**
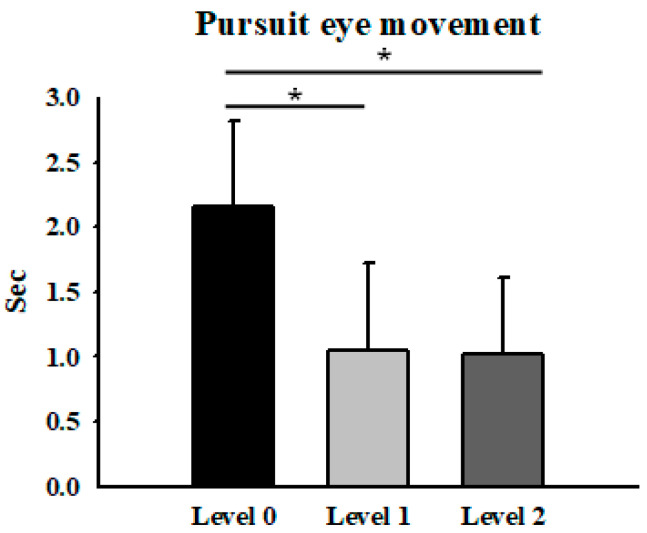
Average smooth pursuit traversal time and corresponding derived velocity across difficulty levels. * Means *p* < 0.05.

The mean pursuit traversal times were 2.16 ± 0.66 s (corresponding to 36.85°/s) at the low level, 1.05 ± 0.67 s (75.81°/s) at the medium level, and 1.02 ± 0.58 s (78.04°/s) at the high level. Significant differences were observed between the low and medium levels, and between the low and high levels (*p* < 0.05) (Figure 4). At higher difficulty levels, frequent catch-up saccades disrupted smooth pursuit, indicating task-induced instability. These results suggest that task difficulty manipulation elicited changes in pursuit performance within this velocity range, potentially reflecting functional limits of stable smooth pursuit in the present task design.

### 3.2. Saccadic Eye Movement Accuracy

**Figure 5:**
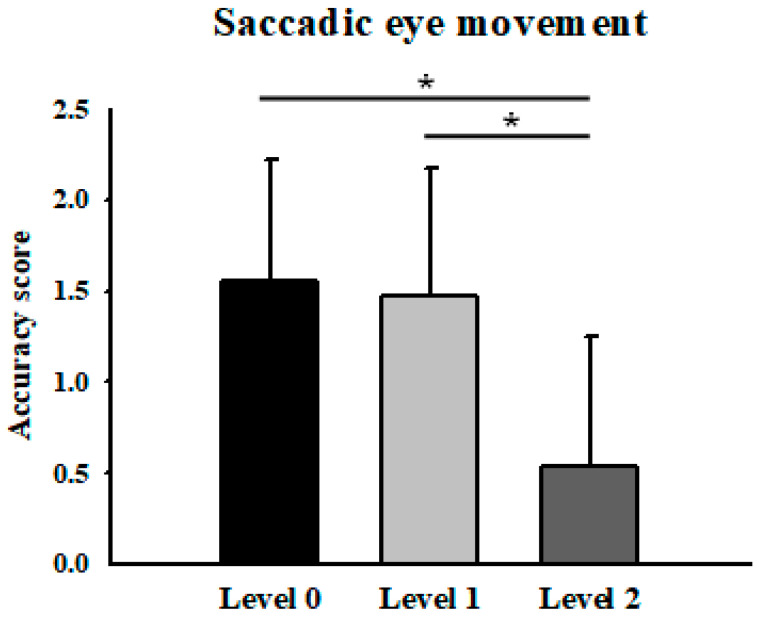
Average accuracy of saccadic eye movement across difficulty levels. * Means *p* < 0.05.

Mean saccadic accuracy scores were 1.55 ± 0.67 (low), 1.48 ± 0.70 (medium), and 0.53 ± 0.71 (high). Significant differences were found between the low and high levels, and between the medium and high levels (*p* < 0.05), indicating reduced accuracy with increasing task difficulty (Figure 5). These findings suggest that task difficulty manipulation resulted in marked changes in saccadic performance across difficulty levels, particularly at higher task demands within the present task design. Given the ordinal nature of the SEM accuracy score, these results should be interpreted as indicative of relative performance trends rather than precise quantitative differences.

The observed decrease in performance at higher difficulty levels suggests that task demands may have exceeded the optimal range of stable ocular motor control, particularly for participants with reduced binocular stability. These findings indicate that task difficulty manipulation within the application effectively differentiated levels of oculomotor performance.

### 3.3. Relationship Between Smooth Pursuit Traversal Time and Phoria

**Figure 6:**
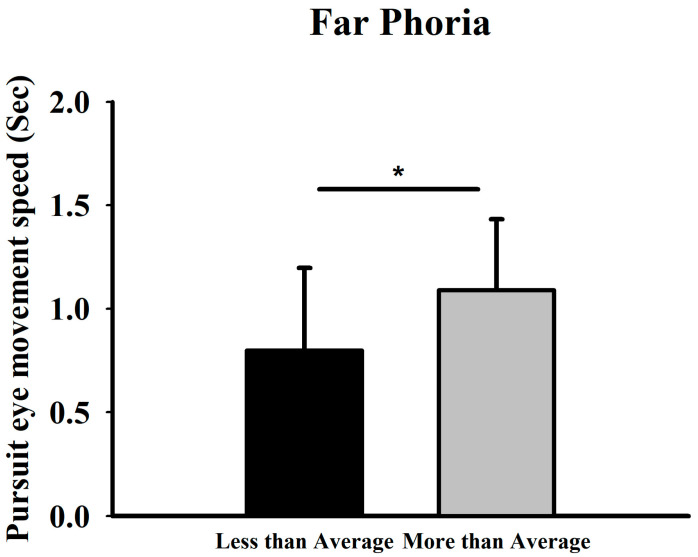
Comparison of smooth pursuit traversal time (and corresponding derived velocity) at high difficulty between groups with above- and below-average far-distance phoria. * Means *p* < 0.05.

Participants were categorized into two groups based on whether their horizontal phoria at distance was above or below the mean value (2.64 ± 1.95Δ). For far-distance phoria, the higher-than-average group showed a longer mean pursuit traversal time of 1.09 s (corresponding to 73.03°/s), while the lower-than-average group showed 0.80 s (99.50°/s) (Figure 6).

**Figure 7:**
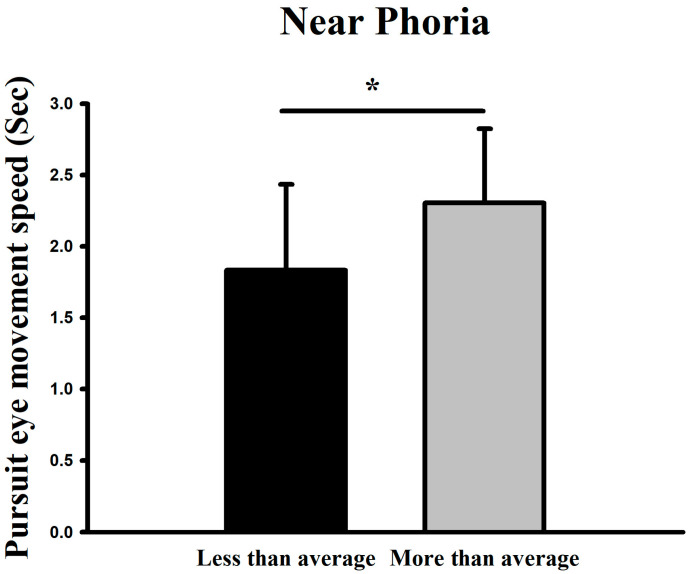
Comparison of smooth pursuit traversal time (and corresponding derived velocity) at low difficulty between groups with above- and below-average near-distance phoria. * Means *p* < 0.05.

For near-distance phoria, the higher-than-average group exhibited a mean velocity of 2.30 s (34.61°/s), while the lower-than-average group showed 1.83 s (43.50°/s). In both far- and near-distance conditions, Participants with higher phoria values exhibited relatively slower pursuit performance under certain task conditions (*p* < 0.05) (Figure 7).

### 3.4. Relationship Between Smooth Pursuit Traversal Time and Divergence Reserve

**Figure 8:**
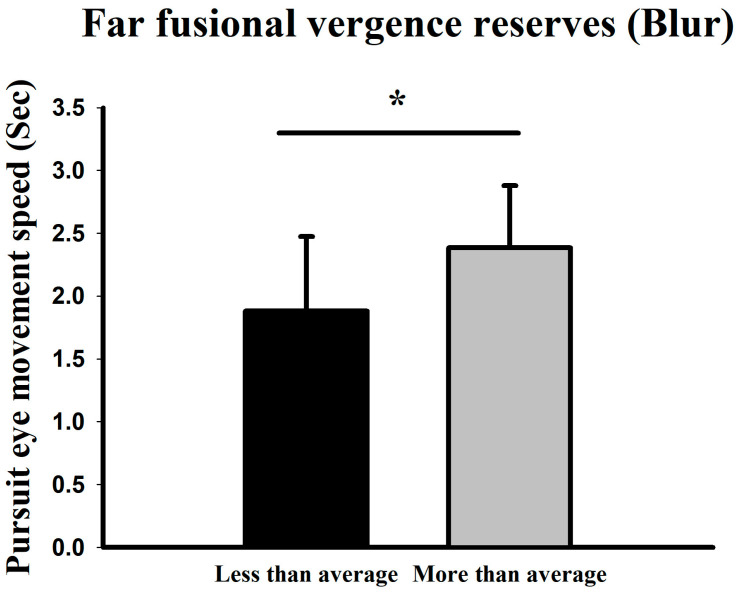
Comparison of smooth pursuit traversal time (and corresponding derived velocity) at low difficulty between groups with above- and below-average far-distance divergence blur points. * Means *p* < 0.05.

Based on the mean far-distance divergence blur point (6.15 ± 6.24Δ), the higher-than-average group exhibited a mean pursuit velocity of 2.38 s (33.45°/s), while the lower-than-average group showed 1.88 s (42.34°/s) (Figure 8).

**Figure 9:**
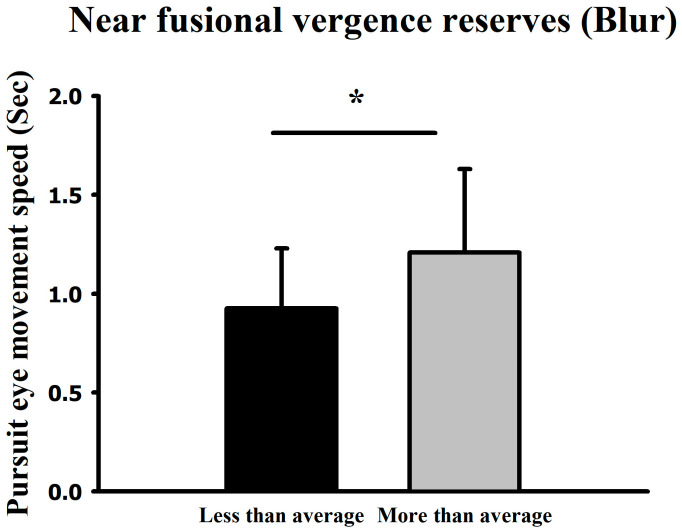
Comparison of smooth pursuit traversal time (and corresponding derived velocity) at medium difficulty between groups with above- and below-average near-distance divergence blur points. * Means *p* < 0.05.

Using the mean near-distance divergence blur point (9.23 ± 4.56Δ) as a reference, the higher-than-average group recorded a mean velocity of 1.21 s (65.79°/s), whereas the lower-than-average group recorded 0.93 s (85.59°/s). In both far- and near-distance conditions, higher blur-point groups demonstrated slower pursuit velocities, showing statistically significant differences (*p* < 0.05) (Figure 9).

### 3.5. Relationship Between Smooth Pursuit Traversal Time and Convergence Break Point

**Figure 10:**
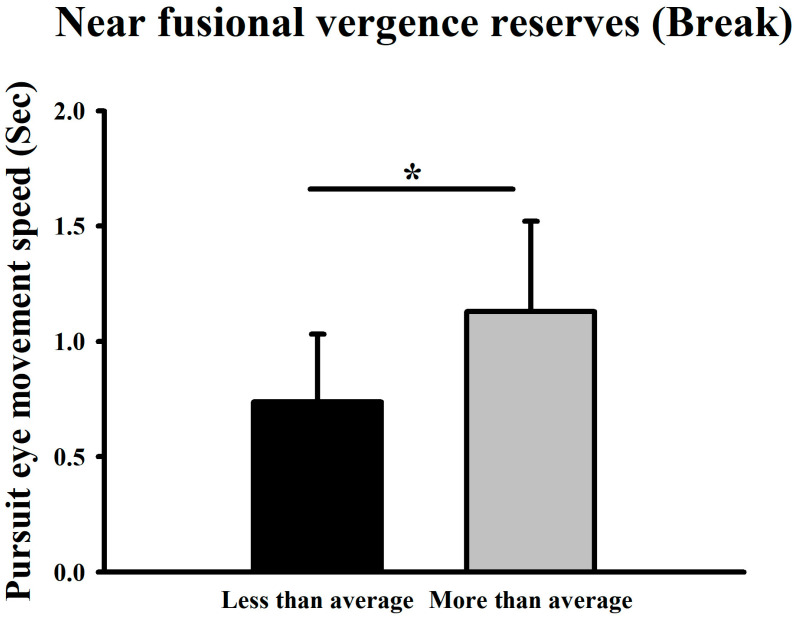
Comparison of smooth pursuit traversal time (and corresponding derived velocity) at medium difficulty between groups with above- and below-average near-distance convergence break points. * Means *p* < 0.05.

When participants were divided according to the mean near-distance convergence break point (15.33 ± 5.75Δ), the higher-than-average group showed a slower mean velocity of 1.13 s (70.44°/s) than the lower-than-average group (0.74 s, 107.57°/s), revealing a statistically significant difference (*p* < 0.05) (Figure 10).

### 3.6. Relationship Between Saccadic Accuracy and Convergence

**Figure 11:**
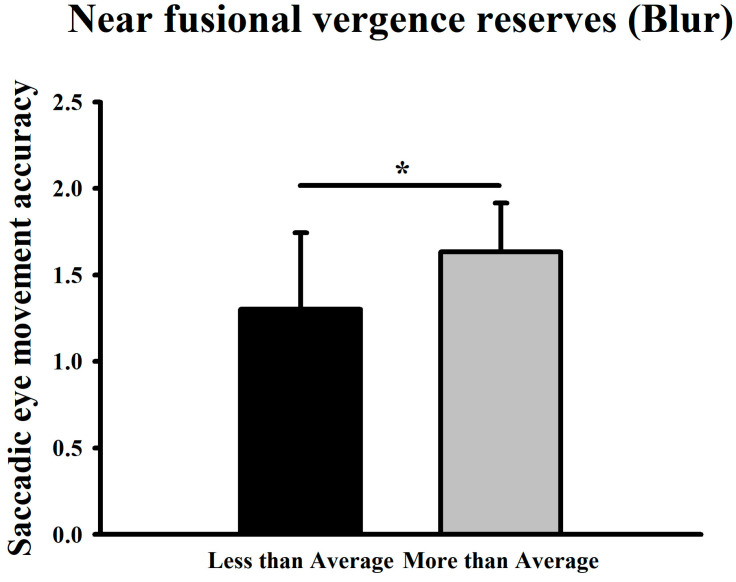
Comparison of saccadic eye-movement accuracy at high difficulty between groups with above- and below-average near-distance convergence blur points. * Means *p* < 0.05.

Based on the mean near-distance convergence blur point (9.24 ± 4.56Δ), the higher-than-average group achieved a mean accuracy score of 1.63, compared to 1.30 in the lower-than-average group, indicating a statistically significant difference (*p* < 0.05) (Figure 11).

In most remaining visual function comparisons, participants with higher-than-average visual function test scores tended to exhibit greater saccadic accuracy; however, these differences were not statistically significant. These findings indicate that tablet-based eye movement assessment may be sensitive to normal inter-individual functional variability, rather than serving as a tool for identifying clinical abnormalities.

## 4. Discussion

This study suggests that a tablet PC–based eye movement assessment system may be capable of capturing functional characteristics of smooth pursuit and saccadic eye movements under graded task conditions. The significant increase in average SPEM velocity across difficulty levels confirmed that the application effectively imposed a graded tracking load. However, the plateauing and slight overshoot of measured velocities at the medium and high levels—exceeding the target speeds—suggest that participants reached the functional limit of pure SPEM, which typically ranges from 40° to 80°/s. The higher apparent velocities likely reflect compensatory high-speed catch-up saccades, indicating a performance ceiling or threshold for the SPEM system within this range of task difficulty. The present findings indicate that a tablet-based system can distinguish between smooth pursuit and rapid eye movement performance across a range of task difficulties in healthy young adults. Importantly, these results should be interpreted as evidence of functional sensitivity rather than diagnostic ability. In the current experiment, variations in eye movement performance should be interpreted as reflecting normal inter-individual functional variability within a healthy population, rather than indicators of abnormal or pathological visual function. In the present study, terms such as “lower accuracy” or “slower pursuit” are used solely to describe relative differences in performance within a healthy population, rather than absolute oculomotor deficits or pathological findings. All participants were neurologically and ophthalmologically healthy, and no clinical classification was intended.

Grouping participants by horizontal phoria revealed that those with greater phoria exhibited significantly slower pursuit velocities, supporting the hypothesis that increased binocular misalignment requires additional compensatory motor effort to maintain fusion. This finding aligns with the report by Bucci et al. [23], who observed slower pursuit velocities in strabismic patients before surgical correction compared to after alignment, suggesting that ocular misalignment increases motor demand and reduces tracking efficiency. The significant associations between pursuit velocity and horizontal phoria further emphasize the interdependence between binocular alignment and ocular motor control. Individuals with greater phoria values may require increased vergence effort to maintain fusion, thereby allocating fewer neural resources to smooth pursuit execution. This interaction may explain the consistently slower pursuit velocities observed in these participants.

Analysis of pursuit velocity by divergence reserve (blur and break points) showed that participants with higher reserves—at both distance and near—displayed slower pursuit velocities. This may be attributable to delayed target tracking caused by the increased ocular excursion required for maintaining binocular alignment during excessive divergence. This result is consistent with Chrobak et al. [24], who found that individuals with schizophrenia exhibited higher separation and recovery points than healthy controls, resulting in reduced fixation time during tracking. Similarly, the relationship between pursuit performance and divergence or convergence reserves suggests that binocular stability plays a critical role in sustaining efficient tracking, particularly under conditions of increased task demand. These findings align with previous reports indicating that compromised vergence control can adversely affect fixation stability and dynamic gaze behaviors.

Comparative analysis of SEM performance based on near convergence capacity revealed that participants with higher blur points achieved significantly greater saccadic accuracy than those with lower blur points. This finding may reflect the nature of saccadic movements, wherein greater baseline divergence facilitates more precise refixation of moving targets. In contrast to smooth pursuit movements, saccadic eye movements demonstrated a distinct performance profile. The higher saccadic accuracy observed among participants with greater near convergence blur points may reflect enhanced binocular flexibility, facilitating rapid and precise gaze shifts. This distinction highlights the functional specificity of different eye movement systems and suggests that smooth pursuit and saccadic performance may be differentially influenced by underlying visual function parameters. Although exploratory associations between visual function parameters and ocular motor performance were observed within the healthy participant group, the present study does not establish clinical diagnostic validity. All participants were healthy young adults, and no clinical population or reference eye-tracking system was included. Therefore, the observed relationships should be interpreted as preliminary and exploratory.

From an application perspective, the proposed system may be most suitable for preliminary screening, visual function monitoring, and home- or school-based assessment contexts, where portability, ease of use, and low cost are prioritized over high-precision measurement. Potential use cases include early functional screening, monitoring of visual fatigue, and longitudinal observation of assessment-related changes. Validation in real-world clinical or educational settings remains an important direction for future research. Importantly, the present study does not attempt to identify abnormal visual function, but rather to examine whether tablet-based eye movement measures are sensitive to natural functional variability within a healthy population. 

Several limitations must be acknowledged. First, the sample size was small and demographically homogeneous, limiting generalizability. Second, the system was not validated against laboratory-grade eye trackers, and measurement accuracy, randomized difficulty testing, precision, and drift were not quantified. Because the Eyedid SDK does not expose numerical angular accuracy, precision, or drift metrics, the present study cannot provide standard eye-tracking performance indices. As a result, absolute accuracy, precision, and velocity values cannot be interpreted as validated eye-tracking metrics. The findings should therefore be understood as reflecting relative functional variability detected by the tablet-based system, rather than validated measures of oculomotor performance. Third, the absence of a clinical population precludes any conclusions regarding diagnostic or disease-specific applicability. These limitations reflect the pilot nature of the study and highlight the need for future validation studies with larger, more diverse samples and reference systems. The ordinal nature of the SEM accuracy score limits the appropriateness of parametric statistical testing. Future studies should analyze the underlying continuous deviation measures or apply ordinal or nonparametric statistical models. Participant stratification into above- and below-mean groups was performed exclusively for exploratory analysis and visualization. This approach does not imply clinical categorization and should be interpreted cautiously. Statistical analyses were exploratory in nature. Effect sizes and confidence intervals were not systematically reported, and multiple comparisons were conducted without formal correction, increasing the risk of Type I error. Furthermore, it is worth noting that the visual interface design, including the background color and target contrast, may have influenced the oculomotor performance and the system’s tracking accuracy. Excessive brightness or insufficient contrast between the stimulus and the background could potentially lead to increased measurement variability or physiological changes such as pupil size fluctuations. Additionally, factors like chromatic aberration or screen-specific display properties might introduce subtle inconsistencies in eye-tracking data. While this study utilized a standardized interface, future research should systematically investigate the impact of various UI color schemes and ambient lighting conditions to further optimize the reliability of tablet-based oculomotor assessments.

Overall, the results suggest the potential feasibility of the proposed system as a supportive screening and monitoring tool, rather than a validated clinical diagnostic instrument. Nonetheless, future research should include broader age groups, larger sample sizes, stricter control of refractive status, and systematic comparisons between individuals with visual anomalies and those with normative function. Such efforts will improve the method’s sensitivity and reliability across diverse populations, advancing its use for objective visual function assessment and the development of accessible screening and assessment protocols. It may be explored in future studies as a potential supportive tool for future exploratory studies investigating functional visual changes associated with ocular or neurological conditions [25,26,27,28]. Although the system includes task designs that may be adaptable for training purposes, the present study exclusively evaluated assessment feasibility, and no conclusions regarding training effects can be drawn.

## 5. Conclusions

Analysis of pursuit traversal time (with corresponding derived velocity) across difficulty levels in the eye-movement assessment application developed for this study revealed significant differences between the low and medium levels and between the low and high levels. These results suggest task difficulty-dependent changes in ocular motor performance across the tested conditions. Similarly, the average accuracy of saccadic (impulse) eye movements differed significantly between the low and high levels and between the medium and high levels, indicating a comparable threshold for accuracy change.

Comparative analyses between clinical visual function test results and oculomotor performance showed that participants with greater distance and near heterophoria, as well as higher blur and break points in divergence reserves, exhibited significantly longer pursuit traversal times (corresponding to slower derived velocities) than those with lower values. In contrast, saccadic accuracy was significantly higher among participants with greater near divergence blur points.

This pilot study demonstrates the feasibility of a tablet PC-based system for assessing smooth pursuit and saccadic eye movements under graded task difficulty. The observed associations between oculomotor performance and conventional visual function measures suggest that such systems may provide complementary functional information. However, the findings should be interpreted as preliminary, and further validation studies are required before clinical application can be considered. Future studies incorporating larger and more diverse samples, clinical populations, and validation against laboratory-grade eye-tracking systems will be essential to determine the reliability, validity, and practical applicability of tablet-based oculomotor assessment tools.

## Data Availability

The data that support the findings of this study are available from the corresponding author upon reasonable request. Due to privacy and ethical restrictions, not all data can be publicly shared, but anonymized data can be provided for academic and research purposes.
